# Definition of the snoring episode index based on the analyses of snoring parameters and the apnea hypopnea index

**DOI:** 10.1038/s41598-022-10934-1

**Published:** 2022-04-26

**Authors:** Su Geun Kim, Sung-Woo Cho, Jeong-Whun Kim

**Affiliations:** 1 Department of Otorhinolaryngology-Head and Neck Surgery, Seoul National University Bundang Hospital, Seoul National University College of Medicine, 82 Gumi-ro 173rd Street, Bundang-gu, Seongnam, Gyeonggi‑do 13620 South Korea; 2 Department of Otorhinolaryngology Head and Neck Surgery, Seoul National University Hospital, Seoul National University College of Medicine, Seoul, South Korea

**Keywords:** Diseases, Diagnosis, Sleep disorders

## Abstract

Although snoring is the most common subjective symptom in obstructive sleep apnea (OSA), an international consensus on the definition of snoring is lacking. This study aimed to define snoring by analyzing correlations between snoring parameters and the apnea hypopnea index (AHI). We retrospectively analyzed the polysomnography data of patients with OSA. A snoring event was defined when airflow pressure was > 200 microbar. We included four snoring parameters. Snoring percentage was defined as the cumulative time of snoring events divided by total sleep time. A snoring episode was defined as the occurrence of ≥ 3 consecutive snoring events, and the snoring episode index was defined as the number of snoring episodes per hour. The average and longest durations of snoring episodes were also investigated. The study enrolled 5035 patients. Their mean AHI was 26.5/h and the mean snoring episode index was 19.2/h. Although the four snoring parameters showed significant correlations with the AHI, the snoring episode index showed the strongest positive correlation with the AHI (r = 0.741, *P* < 0.001). The snoring episode index may be used as a definition of snoring from the perspective of a highly positive correlation with the AHI.

## Introduction

Sleep-related breathing disorder consists of conditions ranging from simple snoring to severe obstructive sleep apnea (OSA)^1^. The detrimental effects of OSA on sleep architecture are well known. OSA also increases the risk of diabetes mellitus, hypertension, heart failure, and stroke, mainly depending on the apnea hypopnea index (AHI)^2,3^. Snoring is the most common subjective symptom in patients with OSA^4^. Its prevalence has been reported to range between 20 and 40%^5,6^, and more than one-third of people with habitual snoring may not have significant apnea and hypopnea events^7^. Although the AHI, which is most commonly used to diagnose OSA, is systematically well defined, no international consensus exists on the definition of snoring. Hence, various measurement methods and definitions for snoring are used by researchers. Snoring can also be defined in various perspective. For example, it can be defined in terms of symptoms that interfere with the bed partner's sleep, focusing on the intensity of sound, or it can be defined in terms of symptoms reflecting the severity of OSA. Through this study, we tried to find the definition of snoring that can best show correlation with AHI, an important indicator that reflects the severity of OSA.

Other typical symptoms of OSA, such as poor sleep quality and daytime sleepiness, are measured primarily through questionnaires such as the Pittsburgh Sleep Quality Index (PSQI) or Epworth Sleepiness Scale (ESS)^8,9^. However, snoring can be measured using polysomnography (PSG), and hence, it is quantifiable. Various methods for defining snoring have been reported in the literature. As a subjective method, the presence, intensity, and duration of snoring is commonly evaluated using questionnaires administered to patients and bed partners^10,11^ or using visual analog scale (VAS) scores^11^. However, these methods have limitations because questionnaires and VAS scores are inherently subjective.

In order to quantitatively express snoring, several studies on OSA measured the frequency and intensity of snoring using objective methods^12,13^. For example, one study detected vibrations of the trachea using a piezoelectric sensor^14^, and other studies attempted an acoustic analysis of snoring by using a microphone^15–17^. However, an international consensus on quantifying snoring is currently lacking, as are studies on the medical implications of the definition of snoring and its relationship with other PSG parameters, particularly the AHI. The aim of this study was to define snoring by analyzing correlations between snoring parameters, which were calculated using PSG data, and the AHI.

## Methods

### Patients and PSG

We retrospectively analyzed the PSG data of patients who visited the sleep clinic at a tertiary hospital between March 2003 and December 2020. All patients underwent full-night PSG (Embla N 7000, Reykjavik, Iceland) in the sleep laboratory. PSG included electroencephalography, electrooculography, chin and limb electromyography, electrocardiography, nasal pressure transducer and thermistor measurements, chest and abdomen respiratory inductance plethysmography, and pulse oximetry. The PSG recordings and scoring of sleep stages and respiratory events were performed by trained sleep physicians as per the guidelines of the American Academy of Sleep Medicine^18^. An apnea event was detected when the signal dropped below 10% of the reference amplitude for 10 s. The peak signal excursion also dropped by ≥ 90% of the pre-event baseline, as detected using an oronasal thermal sensor or an alternative apnea sensor. A hypopnea event was detected when the signal dropped below 70% of the reference amplitude for 10 s, with a > 3% decrease in blood oxygen saturation from the pre-event baseline, or when the event was associated with an arousal. Sleep stages were also recorded for analysis. Sleep efficiency was calculated as the total sleep time divided by the total recording time. Sleep onset was calculated as the duration from the start of the analysis to the first epoch of sleep. The study protocol was approved by the Institutional Review Board of Seoul National University Bundang Hospital (IRB No. B-2108-700-103), which waived the need for written informed consent because of the retrospective nature of this study. The study was confirmed that all methods were carried out in accordance with relevant guidelines and regulations.

### Acquisition of snoring sounds

Snoring was scored on the basis of the pressure airflow signal and audio recording. Airflow pressure of snoring was detected using a nasal cannula in conjunction with a pressure transducer (Natus® adult nasal pressure cannula, Natus Medical, Pleasanton, CA) during full-night PSG recordings. A piezoelectric sensor (Natus® neurology snore sensors, Natus Medical) was also placed in the tracheal area. Snoring was confirmed when the snoring measured through the nasal transducer was also measured by the piezoelectric sensor. A snoring event was defined when the airflow pressure was > 200 microbar, as detected using a nasal transducer. This threshold was derived by consensus among the authors.

### Definition of snoring parameters

**Figure 1:**
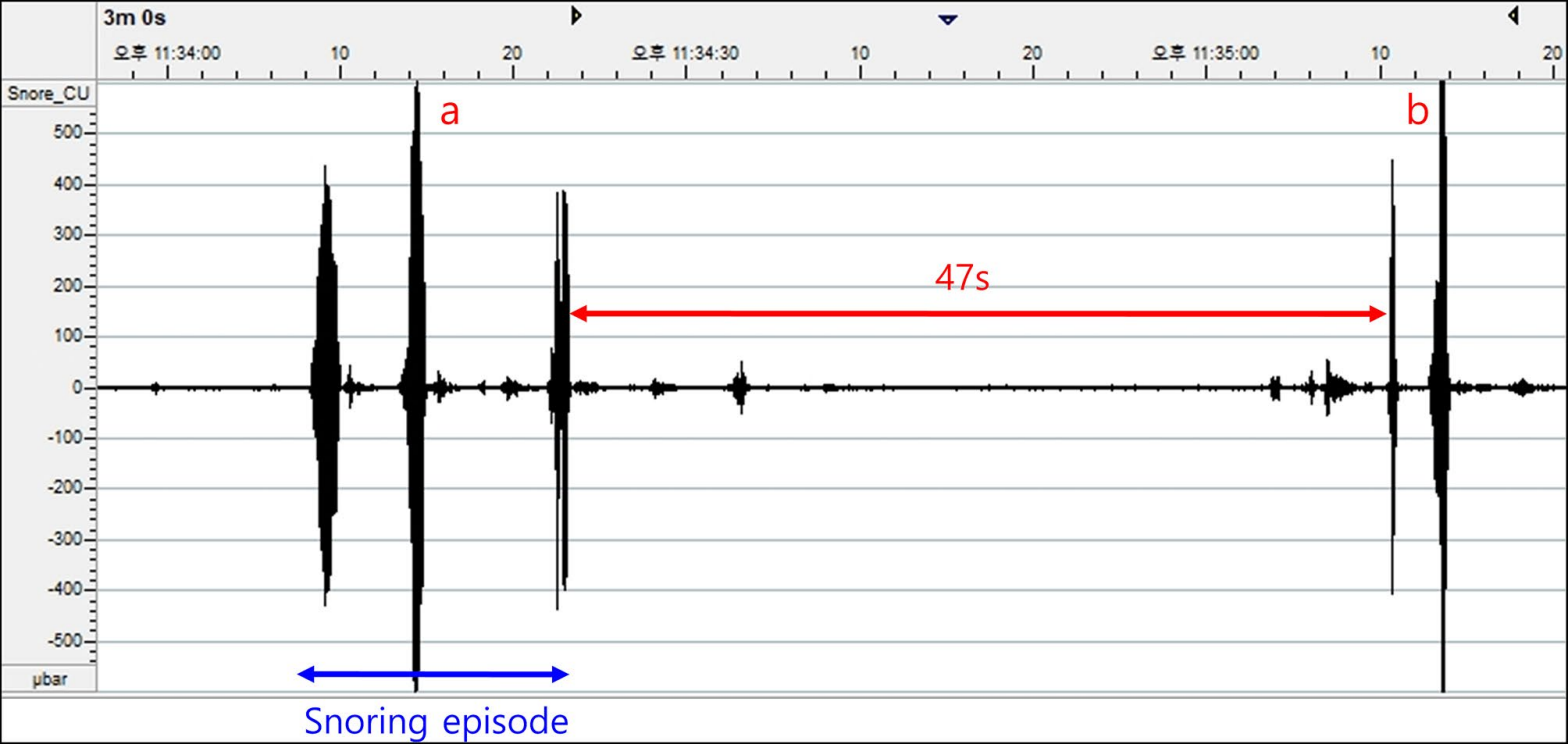
Definitions of a snoring event and snoring episode. When the pressure in the nasal airflow transducer is ≥ 200 microbar, the breath is classified as a snoring event. When there are 3 or more consecutive snoring events, it is defined as one snoring episode as shown in “a”. When 2 consecutive snoring events are separated from each other by > 10 s, they are not counted in the same snoring episode as shown in “b”. The values on the x-axis are in seconds and those on the y-axis are in microbars.

We calculated four different snoring parameters by using the PSG recordings, namely, the snoring percentage, snoring episode index, average snoring episode duration, and longest snoring episode. The snoring percentage was calculated by dividing the cumulative time of snoring events by the total sleep time. A snoring episode was defined as the occurrence of a couple of consecutive snoring events. In addition, to be considered a snoring episode, the interval between the snoring events (between the offset of the previous snoring event and the onset of the next snoring event) had to be within 10 s (Fig. 1). The snoring episode index was defined as the number of snoring episodes divided by the hours of total sleep time.

### Validation of the optimal number of snoring events for a snoring episode

In order to select the optimal number of snoring events for a snoring episode, 12 PSG recordings from 12 patients were randomly selected and reanalyzed. We applied five different definitions of the snoring episode to each PSG recording. The number of snoring events included in the five different definitions of a snoring episode was 1, 2, 3, 4, and 5, respectively. Accordingly, five different snoring episode indices were calculated for each patient. Correlations between each snoring episode index and the AHI were analyzed.

### Statistical analysis

All statistical analyses were performed using IBM SPSS Statistics for Windows, Version 20.0 (IBM Corp., Armonk, NY). Statistical significance was set at *P* < 0.05, and all tests were two-tailed. Categorical variables are presented as numbers, and continuous variables are presented as means ± standard deviations. In order to confirm the relationship between sex and snoring parameters, an independent t-test was performed. Multiple regression analyses were used to evaluate the association between the AHI and snoring parameters.

## Results

### General characteristics and PSG findings

A total of 5,035 patients (3,952 males and 1,083 females) were enrolled in this study. Their mean age was 50.6 ± 13.8 years (range, 8–93 years). Their mean body mass index (BMI) was 26.0 ± 4.1 kg/m^2^ and mean AHI was 26.5 ± 22.7/h. The demographic and PSG data of the patients are summarized in Table 1.

**Table 1:** General and polysomnographic characteristics of the patients.

	Mean ± Standard deviation
Sex (male:female)	3952:1083
Age (years)	50.6 ± 13.8
Body mass index (kg/m^2^)	26.0 ± 4.1
Epworth Sleepiness Scale	9.3 ± 4.9
Pittsburgh Sleep Quality Index	7.7 ± 4.1
Sleep latency (min)	17.8 ± 26.2
Sleep efficiency (%)	80.6 ± 12.0
N3 stage percentage (%)	7.6 ± 8.8
Rapid eye movement stage percentage (%)	16.2 ± 6.4
Supine sleep time percentage (%)	64.6 ± 25.9
Apnea–hypopnea index (/h)	26.5 ± 22.7
Mean snoring percentage (/h)	27.1 ± 20.2
Mean snoring episode index (/h)	19.2 ± 14.9
Mean snoring episode duration (min)	0.9 ± 0.7
Mean longest snoring episode duration (min)	13.4 ± 12.7

The mean snoring episode index was 19.2 ± 14.9/h. According to OSA severity, the mean snoring episode index was 6.9 ± 5.9, 11.6 ± 7.5, 17.0 ± 8.9 and 31.5 ± 16.0 in the primary snorers (AHI < 5), mild (5 ≤ AHI < 15), moderate (15 ≤ AHI < 30) and severe (AHI ≥ 30) OSA subgroup, respectively. The mean snoring percentage, longest snoring episode duration, and average snoring episode duration were 27.1 ± 20.2/h, 0.9 ± 0.7%, and 13.4 ± 12.7%, respectively (Table 1).

### Optimal number of consecutive snoring events for a snoring episode

The PSG reanalyses of the 12 patients included in the optimization of the number of snoring events showed that the mean AHI was 20.0/h and mean snoring episode index was 17.0, 13.0, 11.9, 9.1, and 6.3/h for 1, 2, 3, 4, and 5 consecutive snoring events, respectively, in a snoring episode. The correlation analyses revealed that the correlation coefficient between the AHI and each number of consecutive snoring events was 0.846, 0.849, 0.851, 0.819, and 0.742, respectively. The correlation was greatest with 3 consecutive snoring events in a snoring episode. However, the difference in the correlation coefficient between 2 and 3 snoring events was minimal, and to minimize artifacts that could occur when measuring snoring episodes, 3 snoring events were selected to be the optimal number of consecutive snoring events for defining a snoring episode (Table 2).

**Table 2:** Validation of the optimal number of consecutive snoring events in a snoring episode.

Number of consecutive snoring events	Correlation coefficient	*P* value	Mean snoring episode index ± standard deviation (/h)
1	0.846	0.001	17.0 ± 17.3
2	0.849	< 0.001	13.0 ± 15.1
3	0.851	< 0.001	11.9 ± 15.1
4	0.819	0.001	9.1 ± 11.7
5	0.742	0.006	6.3 ± 8.8

### Correlation between snoring parameters

We analyzed the correlations between snoring parameters. For the snoring episode index, the correlation coefficients with the snoring percentage, average snoring episode duration, and longest snoring episode duration were 0.504 (*P* = 0.000), − 0.261 (*P* = 0.000), and 0.082 (*P* = 0.000), respectively. For the snoring percentage, the correlation coefficients with the snoring episode index, average snoring episode duration, and longest snoring episode duration were 0.504 (*P* = 0.000), 0.424 (*P* = 0.000), and 0.694 (*P* = 0.000), respectively. The correlation coefficient between the average snoring episode duration and the longest snoring episode duration was 0.598 (*P* = 0.000).

### Correlation between snoring parameters and general/PSG parameters

While the snoring percentage showed the highest correlation with the BMI, the other three snoring parameters showed the highest correlation with the AHI among the general and PSG parameters. The snoring episode index showed a highly positive correlation with the AHI (r = 0.741, *P* < 0.001), whereas the average snoring episode duration and longest snoring episode duration were negatively correlated with the AHI (r = − 0.414, *P* < 0.001 and r = − 0.0125, *P* < 0.001, respectively). The relationship between the snoring parameters and PSG parameters is summarized in Table 3. The association between the AHI and the snoring episode index was analyzed after adjusting for age, sex, BMI, snoring percentage, average snoring episode duration, and longest snoring episode duration. The multivariate linear regression model showed that the standardized beta coefficient of the snoring episode index for the AHI was 0.802 (*P* < 0.001).

**Table 3:** Correlation between the snoring parameters and polysomnography parameters.

	Snoring percentage (/h)	Snoring episode index (/h)	Average snoring episode duration (min)	Longest snoring episode duration (min)
Age (years)	− 0.074**	− 0.085**	− 0.080**	− 0.097**
Body mass index (kg/m^2^)	0.230**	0.373**	− 0.090**	0.087**
ESS	0.076**	0.174**	− 0.042*	0.015
PSQI	− 0.112**	− 0.128**	0.012	− 0.053**
Sleep latency (min)	− 0.029*	− 0.085**	0.040*	− 0.011
Sleep efficiency (%)	0.112**	0.130**	0.028†	0.107*
Stage 3 NREM (%)	− 0.020	− 0.178**	0.128**	0.091**
Stage REM (%)	0.020	− 0.052**	0.065**	0.075**
Supine sleep time percentage (%)	− 0.033*	0.097**	− 0.056**	− 0.105**
Apnea–hypopnea index (/h)	0.137**	0.741**	− 0.414**	− 0.125**

ESS, Epworth Sleepiness Scale; PSQI, Pittsburgh Sleep Quality Index; NREM, non-rapid eye movement; REM, rapid eye movement.

*P < 0.05, **P < 0.001.

## Discussion

To our best knowledge, although snoring is the most common symptom in OSA, no international consensus exists regarding its objective definition based on PSG results. Our study suggested that the snoring episode index—the first concept we developed as an indicator related to snoring—highly correlated with the AHI. This showed the potential of utilizing the snoring episode index as a definition of snoring related to the severity of OSA. We conducted this study to identify an index that highly correlated with sleep quality or OSA severity by using snoring-related data that can be easily obtained using PSG, and found that the snoring episode index met this purpose.

Many attempts have been made to define snoring both objectively and easily, but most of them have had some limitations. Initially, attempts were made to assess the severity of snoring by administering questionnaires to snorers or their sleep partners. In one such questionnaire study on snorers and their spouses, subjective factors inevitably played a role, as men underestimated their own snoring and women overestimated their spouse’s snoring^11^. Another study aimed to define snoring by using a VAS. The 10 snoring samples were evaluated by 53 evaluators on a 50-step VAS. The results of that study showed high consistency and concordance, but when the peak level was adjusted to exclude the effect of different volumes of each snoring, the concordance was found to be low^19^. Efforts have also been made to objectively define the intensity of snoring. However, owing to the lack of an international consensus on sound intensity for the definition of snoring, different criteria were applied depending on the investigators. For example, 40 dB or 50 dB was used as a loudness threshold to define snoring^12,20^. Efforts have also been made to define snoring according to its frequency. In a study examining the relationship between the frequency of snoring per hour and sleep-related parameters in 74 people, the frequency of snoring per hour and the AHI showed a weak positive correlation. However, the criteria used for defining the frequency of snoring was unclear^21^. In another study, snoring frequency was defined as the percentage of inspiratory breaths during sleep with sound loudness peaks ≥ 40 dB, and snoring intensity was defined as the mean peak inspiratory sound loudness, which showed a moderate positive correlation with the AHI^22^. Nevertheless, in a literature search, we could not find any studies using a definition of snoring that showed as strong a correlation with the AHI as did the newly suggested snoring episode index.

Before using the snoring episode, which has been used conventionally in our PSG laboratory, as a new definition of snoring, we needed to validate the new concept of a snoring episode. In this study, we finally defined 3 consecutive snoring events as one snoring episode and produced a snoring episode index by dividing the total number of snoring episodes by the total sleep time, proving that the snoring episode index very strongly correlated with the AHI. We defined a single snoring event as a > 200-microbar nasal airflow pressure recorded using a nasal pressure transducer. However, this threshold was derived by consensus among the authors since the polysomnography manual stated that the snoring setting value was not validated. We conducted a study to optimize the number of snoring events included in a single snoring episode. The number of consecutive snoring events included in one snoring episode was set to 1, 2, 3, 4, and 5; thereafter, the entire PSG recording was reread to calculate the snoring episode index in each case, and the number of consecutive snoring events with the best correlation with the AHI was set to define the snoring episode. When 2 or 3 consecutive snoring events were included in one snoring episode, the correlation between the snoring episode index and the AHI was the highest. Therefore, we determined that the conventional use of 3 consecutive snoring events for the definition of one snoring episode was reasonable.

In this study, four parameters related to snoring were used, namely, the snoring episode index, snoring percentage, average snoring episode duration, and longest snoring episode duration, and their correlations with several sleep-related factors were analyzed. The BMI, which is closely related to snoring, showed the highest positive correlation with the snoring episode index among the four parameters. The ESS, a questionnaire reflecting daytime sleepiness (one of the symptoms that indirectly reflects the quality of sleep), also showed the highest positive correlation with the snoring episode index. Meanwhile, the PSQI, a comprehensive survey on sleep quality, showed a negative correlation with the snoring episode index, indicating that it was difficult to accurately predict sleep quality on the basis of snoring. Other major PSG parameters, such as sleep latency, sleep efficiency, rapid eye movement sleep percentage, and supine sleep time percentage, did not show high correlations with snoring parameters.

Establishing a common definition of snoring is important both for research and clinical purposes. Since sleep events such as apnea, hypopnea, oxygen desaturation, and arousal are systematically defined, all researchers can conduct research and interpret research results according to the common definitions. In addition, it is possible to interpret the clinical meaning and explain it to the patient according to the common definitions in the actual medical situation. On the other hand, even though snoring is an important symptom that interferes with a bed partner's sleep quality, and it is an important sleep event in OSA, the definition used differs depending on the researcher or sleep laboratory. Therefore, there is room for confusion when interpreting research results related to snoring or explaining the degree of snoring to patients in real world clinical practice. In particular, the definition of snoring that we tried to establish through this study was the definition in terms of correlation with AHI. The reason is that snoring can be easily measured and can be useful for prescreening OSA. In one study in the United States, 93% of women and 82% of men with moderate to severe OSA were undiagnosed, as were 98% of women and 90% of men with mild OSA^23^. To overcome this issue of underdiagnosis, timely prescreening of OSA is important. If the concept of the snoring episode index presented in this study is included in contactless home sleep tests, the performance of prescreening based on smartphone recording applications may be enhanced. Nevertheless, the snoring episode index proposed in this study has a limitation. The snoring episode index was calculated on the basis of snoring assessed using an airflow pressure sensor. Therefore, it should be validated when snoring is assessed using a smartphone’s sound recording system. In the future, we plan to conduct a prospective study to validate the snoring episode index by utilizing snoring data obtained using sound recording applications on smartphones or artificial intelligence speakers. Although our study showed that the snoring episode index was lowest in the primary snorers, we did not validate how useful this index would be in the population of primary snorers. Future studies are also needed to determine what role the snoring episode index may play in predicting sleep quality and health outcomes in primary snoring.

In conclusion, we suggest a new index for diagnosing snoring, i.e., the snoring episode index, and show that this index highly correlates with the AHI. Although snoring can be defined from various perspectives, the strong correlation between the snoring episode index and OSA severity has a clinical implication given that this index can be used for the prescreening of OSA. Future validation studies using various sound recording systems are required as they will extend the utility of the snoring episode index to home sleep screening applications and devices.

## Data Availability

All the data generated and/or analyzed during the current study are included in this article and are available from the corresponding author on reasonable request.
